# Supplementary material for: Impact of Formulation on the Rheological, Textural, and Sensory Properties of Pistachio Spread
Source: Foods. 2025 Nov 22;14(23):4002. doi: 10.3390/foods14234002 (PMC12692659; doi:10.3390/foods14234002)
Supplement: Supplementary file 1 [file foods-14-04002-s001.zip › foods-3975225-supplementary.pdf]

Table S1. Ingredients (%, w/w) used in the preparation of SP pistachio spread formulations.

Formulation	Pistachio paste	Milk fat	Icing sugar
F1	96	4	-
F2	93	7	-
F3	90	10	-
S1	76	-	24
S2	73	-	27
S3	70	-	30
FS1	66	4	30
FS2	66	7	27
FS3	66	10	24

Table S2. Descriptors used in the sensory evaluation trial of pistachio spreads. Please evaluate the given samples according to the characteristics described below and score them on a 9-point scale (Scoring Scale: 9 = Excellent, 8 = Very good, 7 = Good, 6 = Fairly good, 5 = Moderate, 4 = Fairly poor, 3 = Poor, 2 = Very poor, 1 = Extremely poor).

Attribute	Definition of the Attribute	Evaluation of the attribute
Color	Evaluate the color of the product.	Look at the sample and evaluate the color of the sample.
Flavor	Evaluate freshness, intensity, and the overall intensity of flavor perceived	Smell and taste the sample to assess the aroma strength, freshness, and characteristic pistachio flavor.
Adhesiveness to the spoon	The property of the sample to stick to a surface	Insert a spoon into the sample and lift it slowly to measure stickiness.
Spreadability	Property of the sample to be spread over a surface	Using a spoon, spread the sample onto a plain white sandwich bread and evaluate its spreadability.
Flowability	Ability of the sample to flow or deform under its own weight	Place the spoon in the container and swirl several times to assess fluidity.
Firmness	Resistance of the sample to deformation under applied pressure	Place the sample in the mouth and evaluate the maximum force required to compress it.
Adhesiveness to the mouth	The degree to which the sample sticks to oral surfaces, such as the palate or tongue	Press the sample against the palate with the tongue and evaluate the adhesiveness.
Oily	Perception of oil content and greasy mouth feel	Place the sample in the mouth, swallow, and evaluate the perceived oiliness
Taste	Overall gustatory perception, including sweetness, balance, and aftertaste	Eat the sample and evaluate taste, balance, and aftertaste.
Overall Acceptability	Overall acceptability is a collective score indicating judges' preferences based on sensory attributes	Evaluate the sample as a whole, taking into account all its attributes.
